# Pandemics and People

**DOI:** 10.1128/mSphere.00410-20

**Published:** 2020-05-13

**Authors:** Michael J. Imperiale

**Affiliations:** a University of Michigan Medical School, Ann Arbor, Michigan, USA

## EDITORIAL

As someone who has been involved in discussions about biopreparedness for the better part of the 21st century, albeit for human-made threats, the level of unpreparedness we found ourselves in with respect to the current coronavirus disease 2019 (COVID-19) pandemic has left me especially disappointed. I remember many a meeting inside the Beltway in which various individuals, from academia to government to the private sector, stressed how being prepared for both human-made and natural events is a more apt application of the term “dual use.”

Thinking about this, however, it occurred to me that I should not dwell upon what has happened in the past. Rather, I would like to offer some thoughts on how we can all help each other get through this crisis. For those of you who want a more specific essay on how a laboratory is coping, please read the thoughtful editorial in our sister journal *mBio* by my friend Terry Dermody and his lab members (1).

## 

### Be compassionate and supportive.

It is an understatement to say that the pandemic has added unwanted stress to all our lives. Many of our laboratories have paused research, and those who are performing essential research, including on COVID-19, are having to operate under different safety regulations to maintain social distancing. We are working from home, often with children to care for at the same time. Some of us or our family members have had to take pay cuts, been furloughed, or even been laid off. The scientific enterprise is stressful even in the best of times. When you encounter someone who is struggling, show empathy and understanding.

### Be a mentor.

My experience at the University of Michigan is that the situation is hitting graduate students and postdocs especially hard. They are worried about how this is going to affect their careers moving forward. When will they defend their dissertations? Will people hold apparent gaps in their productivity against them? The latter question is also being asked by junior faculty when thinking about promotion and tenure and even by more senior scientists as they consider grant renewals. When you encounter someone with these kinds of doubts, take the time to counsel them and talk them through their situation.

### Be flexible.

Life often throws us curveballs. Humans are remarkably good at adapting, however, and it encourages me to see the many creative ways people have found to cope. I am not going to try to list them here: all one needs to do is browse social media and the Internet more broadly for examples every day. Our daily research routines have been disrupted, but we can remain engaged. Many of you might be thinking that what life has thrown us this time is more challenging than a Mariano Rivera cut fastball. (Yes, I am a lifelong Yankees fan.) With all due respect to Mariano, we can hit this one!

### Be active.

One of the things I have always noticed is how much walking I do on an average day. My lab is about 100 meters from our department office, to and from which I walk multiple times each day, and I am often travelling between buildings on campus. Working from home has certainly changed that. Make a conscious effort to get outside. Walk, bike, run, do whatever you can that can be accomplished with appropriate social distancing. Your physical and mental health depend on it.

### Be inclusive.

Diverse opinions are important even during “normal” times, but I think they are all the more critical as we try to work together through this crisis. People, especially in leadership positions, are being asked to make many decisions in a compressed time frame. What better time to use the collective mind to inform how we operate? Many of you have been thinking, or are beginning to think, about how to restart your laboratory activities. Engage all members of the group in devising a means to operate that is safe while still allowing projects to resume. Reach out to your colleagues, locally and at other institutions, for ideas.

### Be patient.

It goes without saying that we are going to be living and working differently for a long time. To paraphrase Tony Fauci, the virus will decide when we can move back to the way we are used to. Let us not rush the process of returning to normal. There are lessons to be learned from the 1918 influenza pandemic, during which communities that were cautious in returning to normal suffered less in the long run, in terms of both physical and economic health.

### Be a source of trusted information.

As you know, in our world of 24/7 news and social media, there is a lot of misinformation floating around. Take every opportunity you have, as informed citizens, to educate your families, neighbors, and the broader population about the biology of the virus and how it causes disease, how public health measures work, how diagnostics, therapeutics, and vaccines are developed and tested, and how peer review works. This is but a short list of the areas in which we must engage as scientists who are trying to serve the public with our research.

I am confident that the scientific and health care communities will help to lead us out of the quandary in which we currently live. Let us work to do our parts, individually and collectively, to help out however we can. Stay safe, and stay well.
